# Supplementary material for: RNA-TorsionBERT: leveraging language models for RNA 3D torsion angles prediction
Source: Bioinformatics. 2025 Jan 8;41(1):btaf004. doi: 10.1093/bioinformatics/btaf004 (PMC11758789; doi:10.1093/bioinformatics/btaf004)
Supplement: btaf004_Supplementary_Data [file btaf004_supplementary_data.zip › 8266f_TorsionalAnglesSupplementaryBioinformatics.pdf]

RNA-TorsionBERT: leveraging language models for RNA 3D torsion angles prediction

Clément Bernard, Guillaume Postic, Sahar Ghannay, Fariza Tahi

January 3, 2025

Datasets

Figure S1 shows the distribution of the ribose sugar ring angles for the different datasets used. Their distributions seem quite close, which is also the case for the pseudrotation phase P angle.

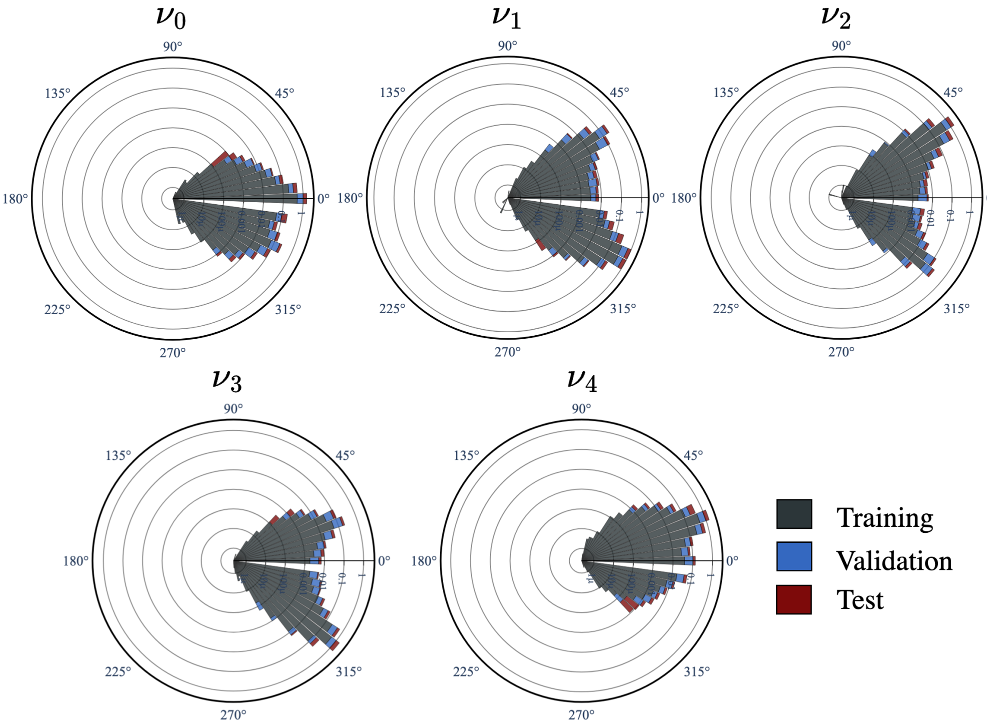

Figure S1: Polar distribution of the five ribose sugar ring angles (ν_0, ν_1, ν_2, ν_3 and ν_4) for the Training, Validation and Test datasets. For each angle, the logarithm of the normalized count is depicted

Experimental protocol

We have fine-tuned both DNABERT [1] and RNABERT [2] for the prediction of torsional and pseudo-torsional angles. For the two models, we used a batch size of 10, the Mean Average loss

with a learning rate of 1e-4 and a weight decay of 0.01. We used the AdamW [3] optimizer. All inputs were padded to have a fixed size of 512 for DNABERT and 440 for RNABERT (limited by the model), and we trained the models for a maximum of 20 epochs. As there is no RNA of sequence length between 440 and 512, we used the same datasets for both RNABERT and DNABERT.

Performances

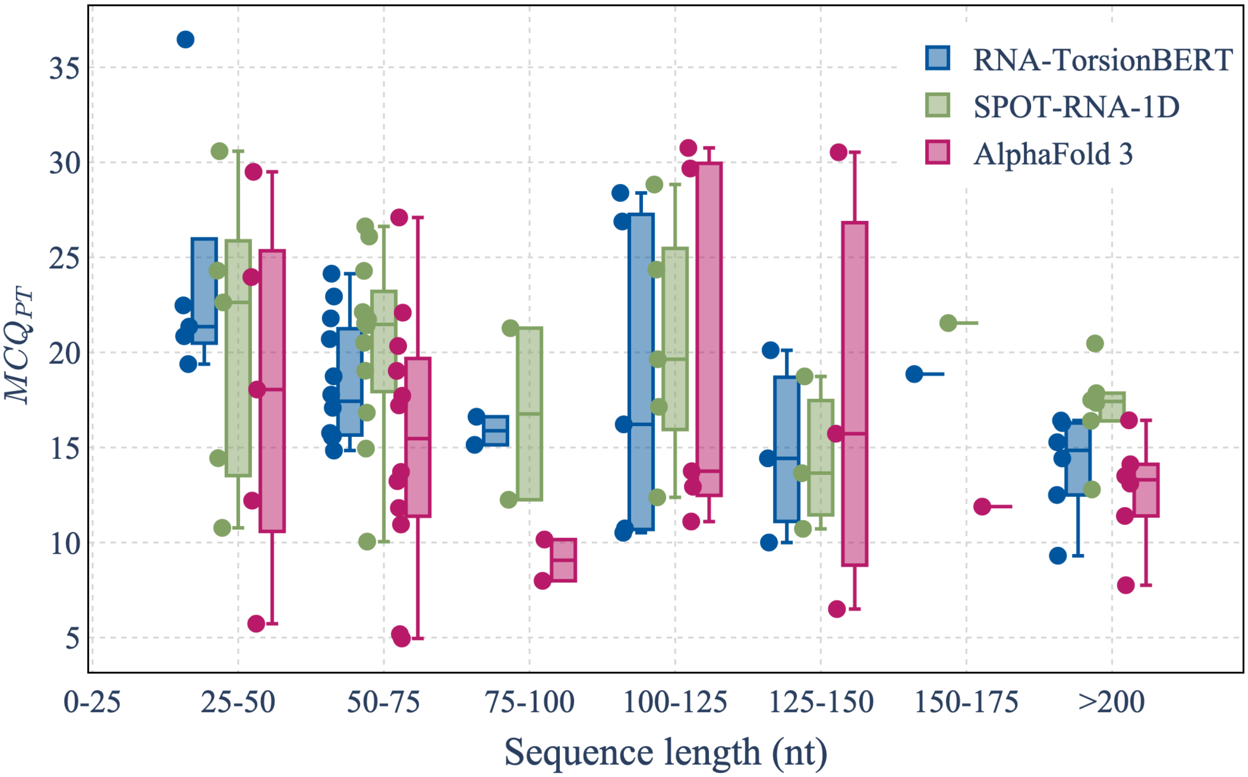

Figure S2: MCQ_{PT} per window of 25nt (from 25nt to 200nt) for RNA-TorsionBERT, SPOT-RNA-1D and AlphaFold 3 inferred angles for the Test set.

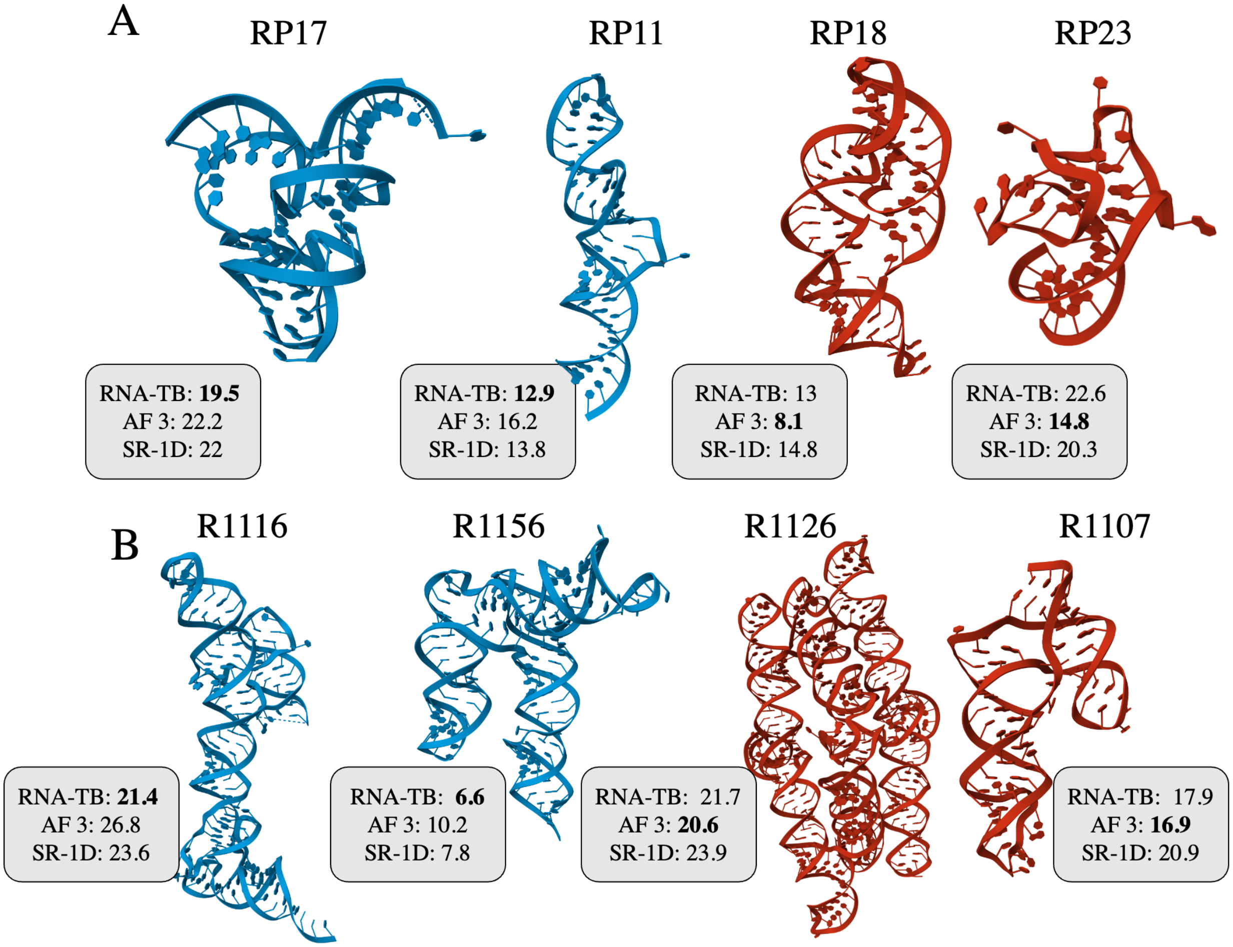

Figure S3: Structures with the associated MCQ for RNA-Puzzles (A) and CASP-RNA (B). In blue are reported examples of structures where RNA-TorsionBERT outperforms AlphaFold 3 and SPOT-RNA-1D. In red are examples of RNA structures where AlphaFold 3 outperforms RNA-TorsionBERT and SPOT-RNA-1D.

Table S1: MCQ per pseudo-torsional angle and MCQ_{PT} (MCQ computed for all the pseudo-torsional angles) over the Test set for RNA-TorsionBERT compared to SPOT-RNA-1D [4]. We also include inferred torsional angles from state-of-the-art methods that predict RNA 3D structures from State-of-the-RNArt [5]. Methods are sorted by MCQ_{PT}.

Models	$MCQ_{(\eta)}$	$MCQ_{(\theta)}$	MCQ_{PT}
RNA-TorsionBERT	15.2	20.8	18.0
SPOT-RNA-1D [4]	17.0	21.3	19.1
AlphaFold3 [6]	**13.8**	**17.4**	**15.6**
IsRNA1 [7]	18.9	26.1	22.4
RNAJP [8]	20.3	25.5	22.8
Vfold-Pipeline [9]	21.0	27.6	24.2
RNAComposer [10]	21.0	28.1	24.5
3dRNA [11]	25.5	31.6	28.5
RhoFold [12]	28.1	31.6	29.8
MC-Sym [13]	28.5	32.9	30.6
trRosettaRNA [14]	26.0	36.9	31.3

Table S2: MCQ_{PT} for our method RNA-TorsionBERT, AlphaFold 3 [6] and SPOT-RNA-1D [4] on secondary motifs averaged on the Test set. Secondary motifs are extracted from RNApdbee [15]

Motifs	RNA-TorsionBERT	AlphaFold 3	SPOT-RNA-1D
Single-stranded	36.4	**31.9**	48.4
Loops	31.3	**30**	42.3
Stems	16.3	**15.6**	24.2

Table S3: MCQ per RNA family for the single-stranded structures from RNA-Puzzles [16–19] dataset. The number of times each model outperforms the others is described in parentheses. The models compared are RNA-TorsionBERT, AlphaFold 3 [6] and SPOT-RNA-1D [4].

Family	RNA-TorsionBERT	AlphaFold 3	SPOT-RNA-1D
Aptamer	18.6 (0/3)	**16.4** (**2**/3)	17.5 (1/3)
Riboregulator	**13.0** (**1**/1)	16.2 (0/1)	13.8 (0/1)
Riboswitch	16 (1/11)	**13.9** (**9**/11)	16.6 (1/11)
Ribozyme	**22.6** (**3**/4)	23.0 (1/4)	24.5 (0/4)
Ricin loop	8.5 (0/1)	**6.6** (**1**/1)	10.9 (0/1)
Virus	13.1 (0/2)	**10.3** (**2**/2)	16.5 (0/2)
All	16.8 (5/22)	**15.3** (**15/22**)	17.7 (1/22)

Model quality assessment based on torsional angles

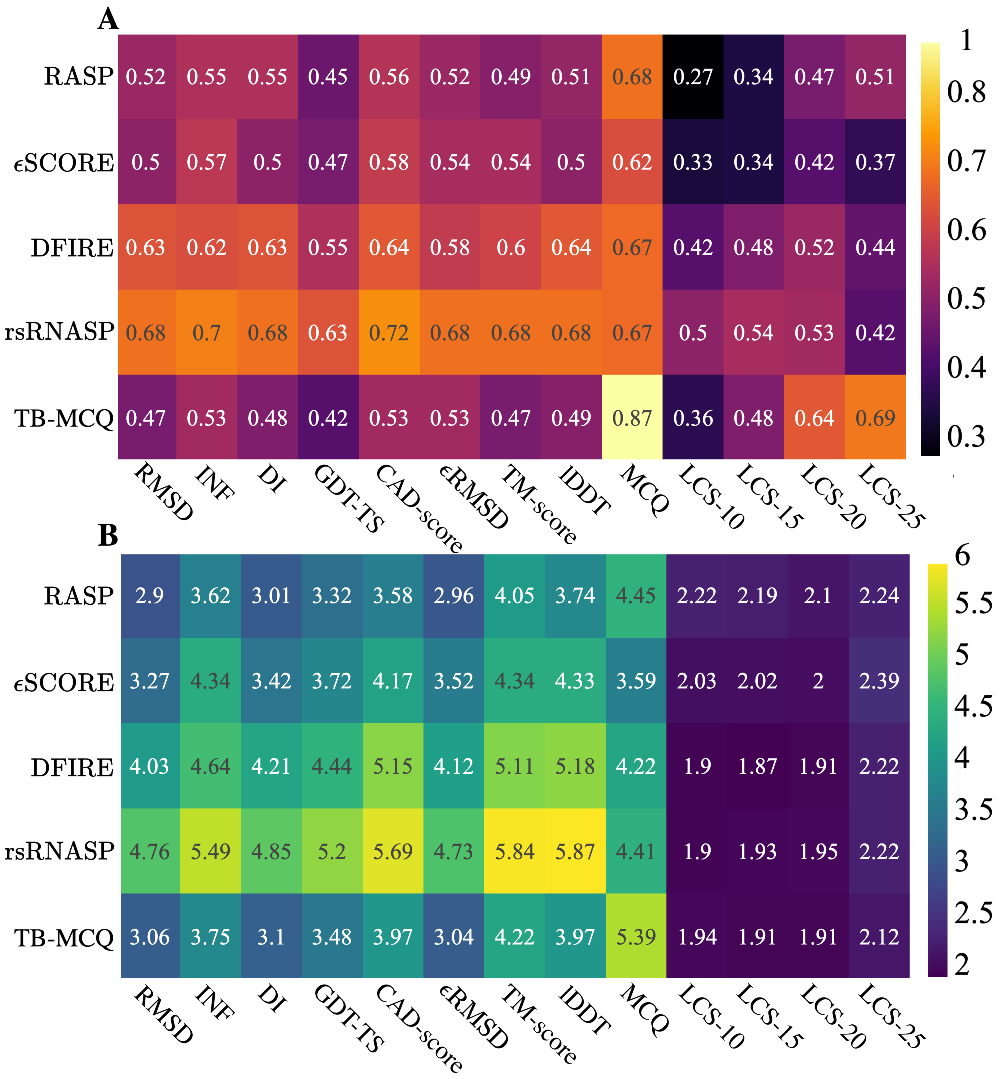

Figure S4: PCC (A) and ES (B) between five different scoring functions (RASP [20], ϵSCORE [21], DIFRE-RNA [22], rsRNASP [23] and our scoring functions TB-MCQ) and ten metrics (RMSD, INF$_{all}$ [24], DI [24], GDT-TS [25], CAD-score [26], ϵRMSD [21], TM-score [27, 28], lDDT [29], MCQ [30], and LCS-TA [31] (with a threshold of 10, 15, 20 and 25)). Values are averaged over the three decoy test sets.

References

1. Ji Y, Zhou Z, Liu H, et al. DNABERT: pre-trained Bidirectional Encoder Representations from Transformers model for DNA-language in genome. Bioinformatics 15 2021;37:2112–20.
2. Akiyama M and Sakakibara Y. Informative RNA base embedding for RNA structural alignment and clustering by deep representation learning. NAR Genomics and Bioinformatics 2022;4:lqac012.
3. Loshchilov I and Hutter F. Decoupled Weight Decay Regularization. arXiv 2019.
4. Singh J, Paliwal K, Singh J, et al. RNA Backbone Torsion and Pseudotorsion Angle Prediction Using Dilated Convolutional Neural Networks. Journal of Chemical Information and Modeling 6 2021;61:2610–22.
5. Bernard C, Postic G, Ghannay S, and Tahi F. State-of-the-RNArt: benchmarking current methods for RNA 3D structure prediction. NAR Genomics and Bioinformatics 2024;6:lqae048.
6. Abramson J, Adler J, Dunger J, et al. Accurate structure prediction of biomolecular interactions with AlphaFold 3. Nature 2024.
7. Zhang D, Li J, and Chen SJ. IsRNA1: De Novo Prediction and Blind Screening of RNA 3D Structures. Journal of Chemical Theory and Computation 3 2021;17:1842–57.
8. Li J and Chen SJ. RNAJP: enhanced RNA 3D structure predictions with non-canonical interactions and global topology sampling. Nucleic Acids Research 7 2023;51:3341–56.
9. Li J, Zhang S, Zhang D, et al. Vfold-Pipeline: a web server for RNA 3D structure prediction from sequences. Bioinformatics 2022;38:4042–3.
10. Popenda M, Szachniuk M, Antczak M, et al. Automated 3D structure composition for large RNAs. Nucleic Acids Research 14 2012;40:e112–e112.
11. Wang J, Wang J, Huang Y, et al. 3dRNA v2.0: An Updated Web Server for RNA 3D Structure Prediction. International Journal of Molecular Sciences 17 2019;20:4116.
12. Shen T, Hu Z, Peng Z, et al. E2Efold-3D: End-to-End Deep Learning Method for Accurate de Novo RNA 3D Structure Prediction. arXiv preprint arXiv:2207.01586 2022.
13. Parisien M and Major F. The MC-Fold and MC-Sym pipeline infers RNA structure from sequence data. Nature 7183 2008;452:51–5.
14. Wang W, Feng C, Han R, et al. trRosettaRNA: automated prediction of RNA 3D structure with transformer network. Nat Commun 2023;14:7266.
15. Zok T, Antczak M, Zurkowski M, et al. RNApdbee 2.0: multifunctional tool for RNA structure annotation. Nucleic Acids Research 2018;46:W30–W35.
16. Cruz JA, Blanchet MF, Boniecki M, et al. RNA-Puzzles : A CASP-like evaluation of RNA three-dimensional structure prediction. RNA 4 2012;18:610–25.
17. Miao Z, Adamiak RW, Blanchet MF, et al. RNA-Puzzles Round II: assessment of RNA structure prediction programs applied to three large RNA structures. RNA 2015;21:1066–84.
18. Miao Z, Adamiak RW, Antczak M, et al. RNA-Puzzles Round III: 3D RNA structure prediction of five riboswitches and one ribozyme. RNA 5 2017;23:655–72.
19. Miao Z, Adamiak RW, Antczak M, et al. RNA-Puzzles Round IV: 3D structure predictions of four ribozymes and two aptamers. RNA 8 2020;26:982–95.

20. Capriotti E, Norambuena T, Marti-Renom MA, et al. All-atom knowledge-based potential for RNA structure prediction and assessment. Bioinformatics 8 2011;27:1086–93.
21. Bottaro S, Di Palma F, and Bussi G. The Role of Nucleobase Interactions in RNA Structure and Dynamics. Nucleic acids research 2014;42.
22. Capriotti E, Norambuena T, Marti-Renom MA, et al. All-atom knowledge-based potential for RNA structure prediction and assessment. Bioinformatics 2011;27:1086–93.
23. Tan YL, Wang X, Shi YZ, et al. rsRNASP: A residue-separation-based statistical potential for RNA 3D structure evaluation. Biophysical Journal 1 2022;121:142–56.
24. Parisien M, Cruz J, Westhof E, et al. New metrics for comparing and assessing discrepancies between RNA 3D structures and models. RNA (New York, N.Y.) 2009;15:1875–85.
25. Zemla A, Venclovas C, Moult J, et al. Processing and analysis of CASP3 protein structure predictions. Proteins: Structure, Function, and Bioinformatics 1999;37:22–9.
26. Olechnovic K, Kulberkyte E, and Venclovas C. CAD-score: A new contact area difference-based function for evaluation of protein structural models. Proteins 2013;81.
27. Zhang Y and Skolnick J. Scoring function for automated assessment of protein structure template quality. Proteins 2004;57:702–10.
28. Gong S, Zhang C, and Zhang Y. RNA-align: quick and accurate alignment of RNA 3D structures based on size-independent TM-scoreRNA. Bioinformatics 21 2019;35:4459–61.
29. Mariani V, Biasini M, Barbato A, et al. lDDT: a local superposition-free score for comparing protein structures and models using distance difference tests. Bioinformatics (Oxford, England) 2013;29:2722–8.
30. Zok T, Popenda M, and Szachniuk M. MCQ4Structures to compute similarity of molecule structures. Central European Journal of Operations Research 2013;22.
31. Wiedemann J, Zok T, Milostan M, et al. LCS-TA to identify similar fragments in RNA 3D structures. BMC Bioinformatics 2017;18:456.
